# Predicting medical graduates’ clinical performance using national competency examination results in Indonesia

**DOI:** 10.1186/s12909-022-03321-x

**Published:** 2022-04-07

**Authors:** Prattama Santoso Utomo, Amandha Boy Timor Randita, Rilani Riskiyana, Felicia Kurniawan, Irwin Aras, Cholis Abrori, Gandes Retno Rahayu

**Affiliations:** 1 Department of Medical Education and Bioethics, Faculty of Medicine, Public Health and Nursing, Universitas Gadjah Mada, Yogyakarta, Indonesia; 2 Medical Education Unit, Faculty of Medicine, Universitas Sebelas Maret, Surakarta, Indonesia; 3 School of Medicine and Health Sciences of Atma, Jaya Catholic University of Indonesia, Jakarta, Indonesia; 4 Faculty of Medicine, Universitas Hasanuddin, Makassar, Indonesia; 5 Faculty of Medicine Universitas Jember, Jember, Indonesia

**Keywords:** National competency examination, Medical students, Medical schools, Predictive, Graduate performance

## Abstract

**Background:**

Indonesia has applied a national competency exit-examination for medical graduates since 2014, called The Indonesia Medical Doctor National Competency Examination (IMDNCE). This examination is administered to ensure the competence of medical graduates from at present 83 medical schools in Indonesia. Although many studies reported their evaluation on medical licensing examinations, there are not many studies performed to evaluate the correlation of a national licensing examination to the graduates’ clinical practice.

**Aims:**

This research aimed to evaluate the performance of new medical doctors in Indonesia in their internship period after the IMDNCE completion, and whether it might become a predictive indicator for the new medical doctors’ clinical performance.

**Methods:**

An observational cross-sectional study was performed in November–December 2017 on 209 doctors who were new medical graduates. Thirty-one senior doctors from a range of regions in Indonesia who were recruited and trained previously participated in the observation. The Clinical Performance Instrument (CPI) tool was developed as an evaluation tool of the new doctors’ clinical competence to be observed for three weeks. The obtained data were analysed using descriptive statistics and correlated to the IMDNCE scores.

**Results:**

The mean (95% CI) of the CPI for all participants was 83.0 (80.8–85.2), with no correlation of CPI score with IMDNCE results in domains of communication, professionalism and patient safety (*p* > 0.05). However, the mean total of the CPI observation scores from doctors who graduated from public medical schools was higher than those graduating from private medical schools. Also, there were differences in scores related to the institution’s accreditation grade (*p* < 0.05).

**Conclusion:**

There is no difference between CPI and national competency examination results. There was no statistical correlation between the clinical performance of new medical doctors during their internship to CBT and OSCE scores in the national competency examination. New doctors’ performance during internship is affected by more complex factors, not only their level of competencies.

## Background

National medical competency examinations have been conducted in many countries to guarantee that the graduating medical doctors are competent based on the required standard. These tests of doctors’ abilities are adminstered to ensure the quality of health-care. Assuring patient safety is one of the main considerations of performing national competency examination [1]. A study in the US indicated that the increased achievement on the national medical examination was correlated with the decrease of patient mortality rate [2]. Hence, developing a standardized national competency examination is very important, especially in countries such as Indonesia that have high mortality rates.

National medical competency examinations have been widely utilised as an evaluation tool in medical education [3–5]. The examination can assess communication, professionalism, patient safety, clinical management, and many other medical skills. National examinations can be used to measure knowledge, skills and attitude of clinical professionalism comprehensively [6], to assess professional development [5], to predict medical doctors’ future clinical performance [7], and also to predict their performance on the subsequent medical training [3, 8]. Norcini et al. [2] also reported that there was a performance difference between doctors who participated in a national licensing examination and those who did not participate.

### National medical competency examination in Indonesia

The Indonesia Medical Doctor National Competency Examination (IMDNCE) is a national medical competency exit exam which has been established since 2014 based on Indonesian Medical Education Act No. 20/2013, which consists of two components. There is the multiple-choice questions using computer-based testing methods (MCQs-CBT) to assess candidates’ knowledge, and an Objective Structured Clinical Examination (OSCE) to assess candidates’ clinical skills performance. The IMDNCE is an important tool, not only to evaluate students’ achievement towards the national standard, but also to evaluate medical graduates’ knowledge/skills, accountability and development [9].

### Medical internship programme in Indonesia

Newly graduated doctors in Indonesia are required to undergo the one-year internship programme. This compulsory programme serves as the first medical practice of the new doctors. During the one-year programme, each new doctor is assigned in a district hospital of the primary health-care facility under the supervision of a senior doctor. New doctors are allowed to conduct independent medical practice after completing the one-year internship programme.

### Rationale

Many studies reported their evaluation of national licensing examinations [3, 4, 8, 10]. However, studies about the effect of a national licensing examination to the subsequent medical practice are scarce. This study aimed to evaluate the performance of new medical doctors in Indonesia in their internship period after the IMDNCE completion. In addition, this study also investigated whether the IMDNCE performance might become a predictive indicator for the new medical doctors’ clinical performance.

## Methods

### Study design and setting

**Fig. 1:**
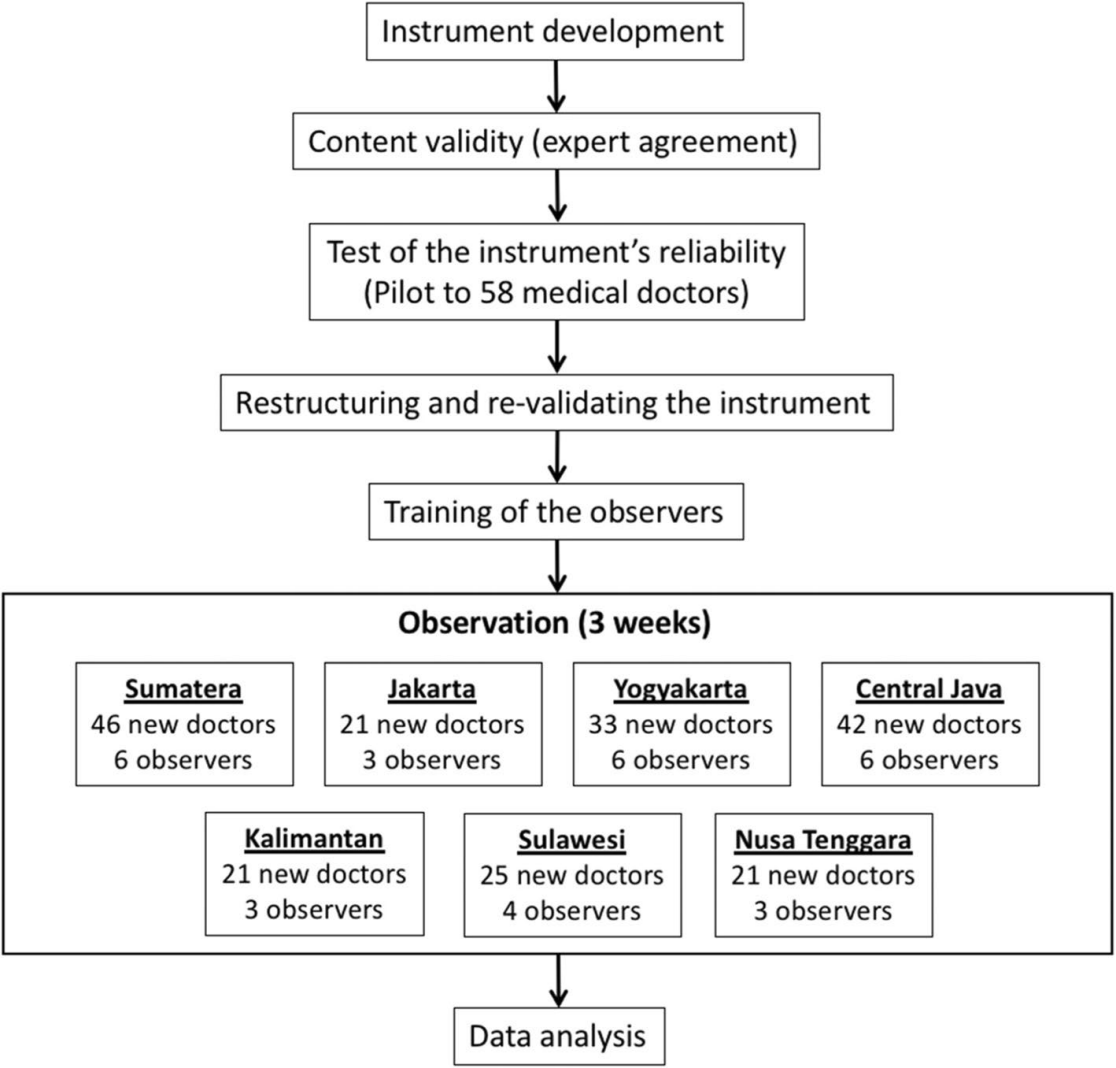
Study protocol

This observational research was a cross-sectional study conducted from November to December 2017 in a range of regions in Indonesia. The details of the study’s protocol are portrayed in Fig. 1.

### Participants

A total of 209 newly graduated doctors who joined the internship program between February 2017 – February 2018 participated in this study. Medical schools in Indonesia are grouped into regions. Hence, purposive sampling was applied to ensure representativeness of each region, schools’ nature (i.e., public or private), and accreditation levels of medical schools (i.e., highest A, B or the lowest C) where the new doctors graduated (see Fig. 1).

In addition, 31 senior doctors from a range of regions in Indonesia were recruited as observers. The observers were trained how to observe the new doctors’ clinical performance using a validated instrument. The observation was conducted for a three-weeks period. The observers were requested to conduct clinical performance evaluation at least once-a-week for each of the new doctors to obtain three observation reports throughout the observation period. The observation was conducted in a range of clinical settings, such as outpatient clinic, emergency room, in-patient setting and field visit service. The observers were provided with information about the general purpose of the study, but were blinded to the characteristics of the interns to avoid possible biases.

### Instrument

This study applied the Clinical Performance Instrument (CPI) which was based on the IMDNCE OSCE examination rubric. There are seven domains in the examination, which are anamnesis, physical examination, supportive examination, clinical diagnosis and differential diagnosis, patient management (pharmacology and non-pharmacology), health education, and professionalism. Discussion with an expert panel was conducted during CPI development to conclude the three domains of CPI. The CPI then underwent a validity study towards 58 medical doctors who practice in primary care setting. Using Likert scale for each items, the medical doctors completed in the instrument based on their perception and experience during clinical practices. The instrument showed a good reliability score (Cronbach-alpha = 0.93) [11].

The CPI consists of three domains of competence (i.e., doctor-patient communication/K1, professionalism/K2 and patient safety/K3) which incorporates 25 observation items (see Table 1), in a 4-point scale. Technical skills (i.e., physical examination, procedural skills, clinical reasoning and written communication) fall under patient safety (K3) domain.

**Table 1:** The Clinical Performance Instrument domains and items

Codes	Competences
**K1**	**Doctor-patient communication**
1.1	Doctor performs discussion with the patient and/or the family members regarding any diagnostic test planning
1.2	Doctor giving information to patient and or the family related to clinical funding and diagnostic
1.3	Doctor giving explanation about the treatment (medic and non medical, including benefit and risks) to patient and or the family
1.4	Doctor giving information advantages and disadvantages about the treatment to patient and or the family honestly
1.5	Doctor explain to patient about chance of healing or failed of treatment
1.6	Doctor giving information about the things that should be do and don’t in order to healing process, including infection prevention
1.7	Doctor explains to patient and or the family about the things that should be do for morbidity prevention
**K2**	**Professionalism**
2.1	Able to keep away from harassing the patients and or the family as a clinician (including emotional relationship, sexual, or inappropriate action)
2.2	Able to keeping themselves from expressing beliefs (politics, religion and moral) that can be trigger of conflict of interest
2.3	Doctor consult to the supervisor if he/she didn't know, doubtful, or not skilled
2.4	Doctor always in healthy condition and not influenced of drugs/others that can be impacted on working
2.5	Doctor always in stable mood (not tenses or emotional) during working
2.6	Doctor have appropriate a break time
2.7	Doctor able to working together with other health worker for patient management
**K3**	**Patient safety**
3.1	Doctor pay attention about aseptic and antiseptic principles, sterilization and controlling medical waste when doing invasive procedure
3.2	Doctor asking patient and or the family about allergy or other drugs history before giving treatment that may contradiction with previous illness
3.3	Doctor make sure the clinical examination instruments are in good condition
3.4	Doctor doing medical procedures with considering clinical affect (benefits and risks) to patients
3.5	Doctor decide medical procedure with considering urgent cases (comply the rule of emergency triage)
3.6	Doctor doing initial treatment before referring the patient
3.7	Doctor doing appropriate referral procedure
3.8	Doctor identify and report if there is a mistake
3.9	Doctor giving advice in order to prevent/avoid medical error
3.10	Doctor implement infection prevention risk principle in health care (ex: management of infectious diseases such as Hepatitis B, Tuberculosis, HIV, etc.)
3.11	Doctor doing invasive procedure in right patient and location (right verification or sign)

### Data analysis

The obtained data were analysed using descriptive statistics to measure mean, median and standard deviations of the three-weeks observations scores. The CPI scores were analysed based on the medical schools’ status and accreditation using the Mann–Whitney and Kruskall-Wallis tests.

The CPI observation scores were subsequently tested using correlation and multiple linear regression analyses against the IMDNCE scores (computer-based test/CBT and OSCE), to measure the predictive value. Multiple linear regression was capable of measuring the relationship between two independent variables, for this instance the IMDNCE score as predictor of CPI (significant if *p* value < 0.05).

### Ethical considerations

This study was ethically approved by the Committee of Research Ethics, Faculty of Medicine Universitas Sebelas Maret (No: 1066/XII/HREC/2016). All study participants (i.e., new doctors and observers) had given their consent before the observer training and the three-weeks observation.

## Results

### Demographics

The 209 new doctors were graduated from medical schools with different levels of accreditation (A, B and C), institutional status (private and public) and region of origin. The new doctors were also conducting their internship in a range of regions (Table 2).

**Table 2:** Characteristics of the study participants

Aspect	Number of Subjects	Total
Medical Schools’ Accreditation		
A	119 (56,9%)	209 (100%)
B	75 (35,9%)	
C	15 (7,2%)	
Medical Schools’ Status		
Public	113 (54,1%)	209 (100%)
Private	96 (45,9%)	
Medical Schools’ Region of Origin		
Sumatera	46 (22,0%)	209 (100%)
Greater Jakarta	33 (15,8%)	
Western Java	3 (1,4%)	
Central Java and Kalimantan	78 (37,3%)	
Eastern Java, Bali, Nusa Tenggara and Sulawesi	25 (12,0%)	
Maluku and Papua	24 (11,5%)	
Internship Area		
Jakarta	21 (10,0%)	209 (100%)
Sulawesi	25 (12,0%)	
Nusa Tenggara	21 (10,0%)	
Sumatera	46 (22,1%)	
Kalimantan	21 (10,0%)	
Central Java	42 (20,1%)	
Yogyakarta	33 (15,8%)	

### CPI observation scores

The mean (95% CI) of the CPI for all participants was 83.0 (80.8–85.2). The mean score for each domains of CPI, there were communication 80.5 (77.2–83.7), profesionalism 93.9 (92.4–95.4), and patient safety 77.8 (74.4–81.1). The mean (95% CI) total of the CPI observation scores of new doctors graduated from public medical schools were better than those graduated from private medical schools, 85.3 (82.6–88.1) vs 80.3 (76.8–83.1) with *p* = 0.044. Moreover, new doctors graduated from A-accredited medical schools performed better than those graduated from B and C-accredited medical schools with mean (95% CI) total CPI score A vs B vs. C: 86.0 (83.3–88.7) vs 79.2 (75.2–83.2) vs 78.8 (70.2–87.5), *p* = 0.002. The results of CPI observation scores are summarised in Table 3.

**Table 3:** CPI observation results based on medical schools’ status and accreditation

Observation Component	Institution Status					Institution Accreditation						
	**Public**		**Private**		**p**	**A**		**B**		**C**		**p**
	x̄ (95% CI)	Me	x̄ (95% CI)	Me		x̄ (95% CI)	Me	x̄ (95% CI)	Me	x̄ (95% CI)	Me	
Communication	82.8 (78.8–86.9)	95.2	77.6 (72.3–82.9)	88.1	0.177	82.8 (78.8–86.9)	95.2	79.1 (73.6–84.6)	85.7	68.3 (50.6–85.9)	76.2	0.164
Professionalism	93.3 (91.1–95.6)	100.0	94.5 (92.5–96.6)	100.0	0.722	95.8 (94.1–97.5)	100.0	91.3 (88.3–94.3)	100.0	91.7 (84.8–98.7)	100.0	0.020*
Patient Safety	81.8 (77.9–85,7)	87.9	77.9 (67.5–78.4)	81.8	0.023*	81.7 (77.7–85.7)	90.9	71.6 (65.3–77.8)	81.8	77.4 (65.8–88.9)	81.8	0.010*
Total	85.3 (82.6–88.1)	92	80.3 (76.8–83.1)	85.3	0.044*	86.0 (83.3–88.7)	93.3	79.2 (75.2–83.2)	84.0	78.8 (70.2–87.5)	81.3	0.002*

*x̄* Mean, *95% CI* 95% confident interval, *Me* Median

Furthermore, the CPI observation scores were tested against the IMDNCE CBT and OSCE scores of the corresponding domains (i.e., namely communication, professionalism and patient safety). There were no correlations between the CPI observation scores to IMDNCE CBT nor OSCE scores (*p* = 0.368 – 0.928) as outlined in Table 4.

**Table 4:** Correlation of CPI observation results and INMCDE CBT and OSCE scores

INMCDE Results	CPI Observation Results							
	Communication		Professionalism		Patient Safety		Total	
	p	R^2^	p	R^2^	p	R^2^	p	R^2^
CBT								
Communication	0.871	-0.013	0.903	0.010	0.686	0.032	0.689	0.031
Professionalism	0.740	-0.026	0.688	-0.032	0.438	0.061	0.677	0.033
Patient Safety	0.854	0.014	0.779	-0.022	0.897	-0.010	0.928	-0.007
Total	0.783	0.019	0.841	-0.014	0.556	0.041	0.546	0.042
OSCE								
Communication	0.487	-0.048	0.500	-0.047	0.461	0.051	0.885	0.010
Professionalism	0.031	-0.150	0.061	-0.130	0.907	-0.008	0.368	-0.063
Patient Safety	0.898	-0.009	0.185	-0.093	0.434	-0.055	0.529	-0.044
Total	0.656	-0.031	0.088	-0.119	0.561	-0.041	0.541	-0.043

## Discussion

IMDNCE serves as a means of standardization as well as quality assurance of medical school graduates in Indonesia. As a standardized exit examination, IMDNCE is expected to be able to predict the performance of fresh graduate doctors in their early phase of clinical practice, especially during the internship [1, 12, 13]. Standardization of graduates through IMDNCE has also been expected to assure the quality of Indonesian doctors. This expectation is manifested in the study result, showing that there was no difference between CBT and OSCE scores and clinical observation results during internship (*p* > 0.05). There were no differences in their performances regarding communication, professionalism, and patient safety competences as well. These results may suggest that through IMDNCE, doctors could demonstrate standardized quality medical care and the test items (CBT and OSCE) could be used as tools to standardize the quality of clinical performance.

National examinations are necessary to assure the quality of doctor candidates within the diversity of medical education institutions [14]. The diversity is influenced by several factors ranging from the input, process, and output of the curriculum. The Indonesian Doctors Standard of Competencies (SKDI) 2012 acts as a national standard for the medical education process with seven areas of competences. However, the implementation of the standard is still influenced by other factors that might affect the quality of medical education in Indonesia, e.g. human resource, learning resources, research development and organization, curriculum development and innovation, and a comprehensive internal quality assurance process. These factors emphasize the need for output standardization through IMDNCE.

Institutional factors are not the only concerns that contribute to the quality of output and the clinical performance of medical school graduates. Internship as the initial phase of clinical practice in real settings for Indonesian medical school graduates might be affected by several factors that are not taught during their educational period [15, 16]. The national health care system with its universal health coverage principal becomes a major influence for clinical performance [1]. The Indonesian health care system prioritizes the strengthening of primary services, whether promotive, preventive, curative, and rehabilitative, in which the intern doctors are at the forefront of primary care. So the phenomenon raises the ratio of patient/doctor by increasing the number of doctors in primary care facilities [17], which, of course, leads to a shift in the quality of care that is affected by the number of patients and the length of service time. This would be different if the phase of education, both pre-clinic and clinics are more ideal both theoretical and clinical practice. Although the professional performance of physicians in this study is not influenced by CBT and OSCE value output, it is necessary to review the efficiency of physician work in terms of implementation of the national health care system, especially in the primary care.

This study identified that the IMDNCE results did not predict new-doctors’ clinical performance scores. The results might be related to the interns’ practice that not yet representing the competences in the Indonesian Doctors Standard of Competences (SKDI) since the IMDNCE items are developed based on SKDI. The new-doctors would not be able to practice in the optimum medical authority stated in SKDI since they were still under the supervision of senior doctors. Moreover, the real clinical setting might differ with what the new-doctors had learnt during their education. For instance, some of the new-doctors were assigned in a district hospital while some of them were in the primary health care centre which had several possible limitations such as funding, facility and the lower doctor-to-patient ratio. The new-doctors might use distinct approaches to cope with the diverse environments [18], which could be different to their approach examined during IMDNCE. Moreover, clinical competence judgement may show a different result when performed under unsimilar administration condition [19]. The disparity between the standards used in IMDNCE and the actual clinical setting makes it complicated to predict the clinical performance based on the IMDNCE results. The level of knowledge and skills are traditionally pertinent to the doctors’ preparedness for practice. However, several factors are argued to influence the performance of doctors in the clinical setting. Doctors shape their conception of professionalism and clinical empathy through the complex cognitive process of role modelling [20]. Meanwhile, mentorship quality in the workplace is argued to influence doctors’ confidence and perception about their own competencies [21]. The individual factors of the novice practitioners during the transitional period, such as the ability to adjust, adapt and manage stress, predispose their clinical performance [22].

Nevertheless, the new-doctors’ clinical performance observed was significantly different for each of the medical schools’ accreditation level. The new-doctors graduated from ‘A’-level accredited medical schools achieved better CPI score than the other levels. This finding corresponds with the evaluation results in 2014–2016 in which IMDNCE participants from higher-accredited medical schools achieved the better score than others from lower-accredited medical schools [23, 24]. Moreover, this result also resonates to another study in USMLE context in which candidates who trained in accredited educational institutions achieved better examination performance where accreditation of educational programmes was associated with the production of more highly skilled physicians [25]. Therefore, this study is capable of providing an evaluation of the educational outcomes at the higher, Level 4A, of the Kirkpatrick’s hierarchy [26] based on graduates’ actual practice observed.

### Limitations

This study showed different results compared to other studies which suggested that national/licensing examination performance predicted performance in the actual clinical practice. Nonetheless, this study included a range of clinical performance aspects such as communication, professionalism and patient-safety for newly-graduated doctors when published studies only focused on single clinical performance [27, 28], or performed after many years of practice [7, 29]. Hence, this study provides new information about short-term performance evaluation following a national competency examination. The instrument used could further validated to ensure construct and concurrent validity, to be used in other settings. Additionally, this study was observational where it might be hard to control the possible confounding factors such as the variety of geographical area in Indonesia. Nevertheless, this study investigated participants from a range of provinces in Indonesia to represent both urban and rural areas which might mitigate the possibility of geographical confoundings. Since cause-effect relationships are difficult to establish even in an experimental research, a more rigorous methodology such as quasi-experiments with control groups would provide wider perspectives.

## Conclusion

There is no difference between CPI and national competency examination results, but there was a significant difference of clinical performace score based on graduates’ medical schools’ nature and accreditation level. There was no statistical correlation between clinical performance of new medical doctors during internship to CBT and OSCE scores in national competency examination. This may suggest that new doctors’ performance during internship is affected by more complex factors, not only their level of competencies. Further cohort studies with longer observation period are recommended to investigate the development of medical graduates’ clinical practice as a follow up to this research.

## Data Availability

Raw data of this study is available upon request to the researchers/authors.

## Abbreviations

IMDNCE: Indonesia Medical Doctor National Competency Examination
SKDI: Standar Kompetensi Dokter Indonesia—The Indonesian Doctors Standard of Competencies
OSCE: Objective Structured Clinical Examination
CPI: Clinical Performance Instrument
